# Effect of repetitive transcranial magnetic stimulation on depression and cognition in individuals with traumatic brain injury: a systematic review and meta-analysis

**DOI:** 10.1038/s41598-021-95838-2

**Published:** 2021-08-20

**Authors:** Ping-Yen Tsai, Yang-Ching Chen, Jia-Yi Wang, Kuo-Hsuan Chung, Chien-Hung Lai

**Affiliations:** 1 School of Medicine, College of Medicine, Taipei Medical University, Taipei, Taiwan; 2 Department of Family Medicine, School of Medicine, College of Medicine, Taipei Medical University, Taipei, Taiwan; 3 Department of Family Medicine, Taipei Medical University Hospital, Taipei, Taiwan; 4 Department of Neurosurgery, Taipei Medical University Hospital, Taipei, Taiwan; 5 Neuroscience Research Center, Taipei Medical University, Taipei, Taiwan; 6 Department of Psychiatry, School of Medicine, College of Medicine, Taipei Medical University, Taipei, Taiwan; 7 Department of Psychiatry and Psychiatric Research Center, Taipei Medical University Hospital, Taipei, Taiwan; 8 Department of Physical Medicine and Rehabilitation, School of Medicine, College of Medicine, Taipei Medical University, Taipei, Taiwan; 9 Department of Physical Medicine and Rehabilitation, Taipei Medical University Hospital, No. 252, Wu-Hsing St., Taipei City, 110 Taiwan

**Keywords:** Biophysics, Neurology

## Abstract

Repetitive transcranial magnetic stimulation (rTMS) is an FDA-approved therapy in major depressive disorder. However, its treatment efficacy on depression after traumatic brain injury (TBI) remains inconclusive. We conducted a meta-analysis to assess the effectiveness of executing rTMS over dorsolateral prefrontal cortex (DLPFC) on depression, cognitive impairment and post-concussion syndrome in individuals with traumatic brain injury. This study contained seven randomized controlled trials that published before April 5, 2020 in PubMed, Embase, Scopus, Cochrane, and Web of Science databases. The rTMS had significant anti-depressant effect (SMD = 1.03, *p* = 0.02), but the effects dissipated at 1-month follow-up (SMD = 0.39, *p* = 0.62). In the subgroup analysis, only applying rTMS to left DLPFC area of post-TBI patients showed significant anti-depressant effect (SMD = 0.98, *p* = 0.04). Moreover, current data observed that rTMS on post-TBI patients possessed substantial improvement in visuospatial memory (SMD = 0.39, *p* < 0.0001), but wasn’t in processing speed (SMD = − 0.18, *p* = 0.32) and selective attention (SMD = 0.21, *p* = 0.31). In addition, the effect of rTMS is not superior to sham on postconcussion syndrome. In conclusion, the short-term antidepressant effect of left DLPFC rTMS in patients with TBI was significant. However, the effectiveness of rTMS on cognition and postconcussion syndrome in patients with post-TBI depression was limited.

## Introduction

Traumatic brain injury (TBI) has been recognized as a global health concern in recent years. Every year, 27–69 million people worldwide experience TBI^1,2^. TBI not only is a major cause of death and disability but also leads to many neurological and psychological sequelae that increase global burden, including depression and cognitive impairment^3,4^. Within the first year after TBI, the prevalence rate of major depressive disorder (MDD) ranges from 25 to 53%^5,6^. From 2007 to 2017, depressive disorder was the third leading burden-causing disease^7^. Cognitive impairment, characterized by alterations in judgment, memory, attention, planning, processing speed, and executive functions, is another complication that limits daily activities following TBI^8^. Approximately 33% of patients with mild TBI have experienced short-term functional impairment, 80% of which has resolved within 6 months^9^. Moderate to severe TBI may lead to persistent impairment in cognitive function at 6 months^10^. Although one-third of patients with severe TBI have normal neuropsychological test performance at 3 months, patients with TBI with cognitive impairment have high future disability risk^10^. Therefore, early therapeutic intervention for post-TBI depression and cognitive impairment has become increasingly critical.

Functional neuroanatomy provides insight into the pathomechanism of depression and cognitive impairment. Left dorsolateral prefrontal cortex (DLPFC) hypoactivity and right DLPFC hyperactivity are involved in depression pathogenesis. Depression severity is associated with right DLPFC hyperactivity^11^. The DLPFC is responsible for emotional judgement as well as cognitive and executive function^12^. The functional connections between the prefrontal cortex and other regions of the brain form different networks, including the default mode network and cognitive control network^13,14^. TBI leads to diffuse axonal injury that disrupts network synchrony^15^. To restore functional connectivity, a functional magnetic resonance imaging-based approach was used to target and modulate specific cortical areas in TBI-induced depression^16^.

Repetitive transcranial magnetic stimulation (rTMS) is a noninvasive neuromodulation approach; in rTMS, the electric current generated by the coil produces a pulsed magnetic field that stimulates underlying cortical neurons. Studies have demonstrated that a high frequency (≥ 5 Hz) of rTMS activates neuronal activity, whereas a low frequency (≤ 1 Hz) suppresses neuronal excitement^17^. Neuronal activity can be modulated through pulse frequency adjustment^18^. For clinical application, rTMS has been proved effective in improving symptoms of several diseases, including Parkinson disease (PD)^19^, Alzheimer disease^20^, stroke^21^, and treatment-resistant depression (TRD)^22^. In the study of Xie et al., rTMS exhibits similar antidepressant effect as selective serotonin reuptake inhibitors in individuals with PD^23^. Moreover, rTMS has significantly better remission rate of depression than sham group, but fails to improve cognitive function in stroke survivors^24^. Thus, rTMS has been proposed for treating people with TBI. Nevertheless, the effect of rTMS on TBI-induced depression or cognitive impairment remains controversial, although related publications have increased from 2018 to 2020. Thus, a systematic review with meta-analysis is crucial to validate the effectiveness of rTMS in treating post-TBI complications. A meta-analysis by Gao et al. revealed that high-frequency rTMS (HF rTMS) over the left DLPFC and low-frequency rTMS over the right DLPFC exhibited similar effects on MDD^25^. However, to our knowledge, no systematic review with meta-analysis has been conducted to ascertain the effectiveness of rTMS on TBI-induced depression or cognitive impairment.

Therefore, the principle aim of this meta-analysis is to determine the efficacy of rTMS over DLPFC in treating TBI-induced depression. Next, our secondary purpose is to investigate whether rTMS can improve cognitive decline and post-concussion syndrome in patients with depression. Finally, current study also evaluates the stimulation parameters of rTMS and the quality of the past studies to make suggestions for future research.

## Methods

### Literature search

Multiple databases, including PubMed, Embase, Scopus, Cochrane, and Web of Science, were searched for randomized controlled trials (RCTs) published before April 5, 2020. The search terms were “traumatic brain injury [mesh]” AND “transcranial magnetic stimulation [mesh].” The meta-analysis was registered as CRD42020200348 in PROSPERO.

### Study selection

Two authors independently screened the titles and abstracts of articles. Then, a full-text review was performed and eligible studies were included. In the case of discrepancy between two reviewers, the conflict was resolved through discussion among the two reviewers and a third reviewer to reach consensus. Studies fulfilling the following criteria were included: (1) patients with TBI, (2) patients with post-TBI depression (Montgomery–Asberg Depression Rating Scale [MADRS] > 6 or Hamilton Rating Scale for Depression [HAMD] > 7) or participants having moderate to severe depression on average in the selected study, (3) age ≥ 18 years, (4) the use of rTMS as intervention (or combined with certain therapy serving as control), and (5) depression evaluated preintervention and postintervention, (6) RCT as study design. We excluded studies that included patients with (1) a history of TMS therapy or (2) a metallic or electronic implant such as a pacemaker.

### Quality assessment

Risk of bias of each included study was assessed using the RoB 2.0 tool. The domains evaluated were risk of bias in (1) the randomization process, (2) intended interventions, (3) missing data, (4) outcome measurement, and (5) selection of the reported result.

### Data extraction and analysis

Reviewers extracted the following data: (1) patient characteristics, (2) number of patients receiving sham or rTMS treatment, (3) study design, (4) outcome measured, (5) rTMS protocol, and (6) standardized mean difference and standard error of change scores after interventions. Since current study used different scores or scales, we combined the effect size by the standardized mean difference, calculated from mean difference divided by standard deviation. Cohen’s d effect size was used in our analysis. Data extracted from the selected studies were analyzed using RevMan 5.3. We used the I^2^ test to examine heterogeneity. Data were synthesized using a random-effects model.

## Results

### Study selection

Through the initial database search, 1467 articles were identified. After removing duplicate studies, 876 articles were obtained, and after further review of titles and abstracts, we obtained 65 articles. The 65 studies were thoroughly reviewed, and most of them were excluded due to their non-RCT design and other outcomes. Finally, seven RCTs that compared the effects of rTMS treatment with sham on post-TBI depression, cognitive impairment, or postconcussion syndrome (PCS) were included (Fig. 1a).

**﻿Figure 1:**
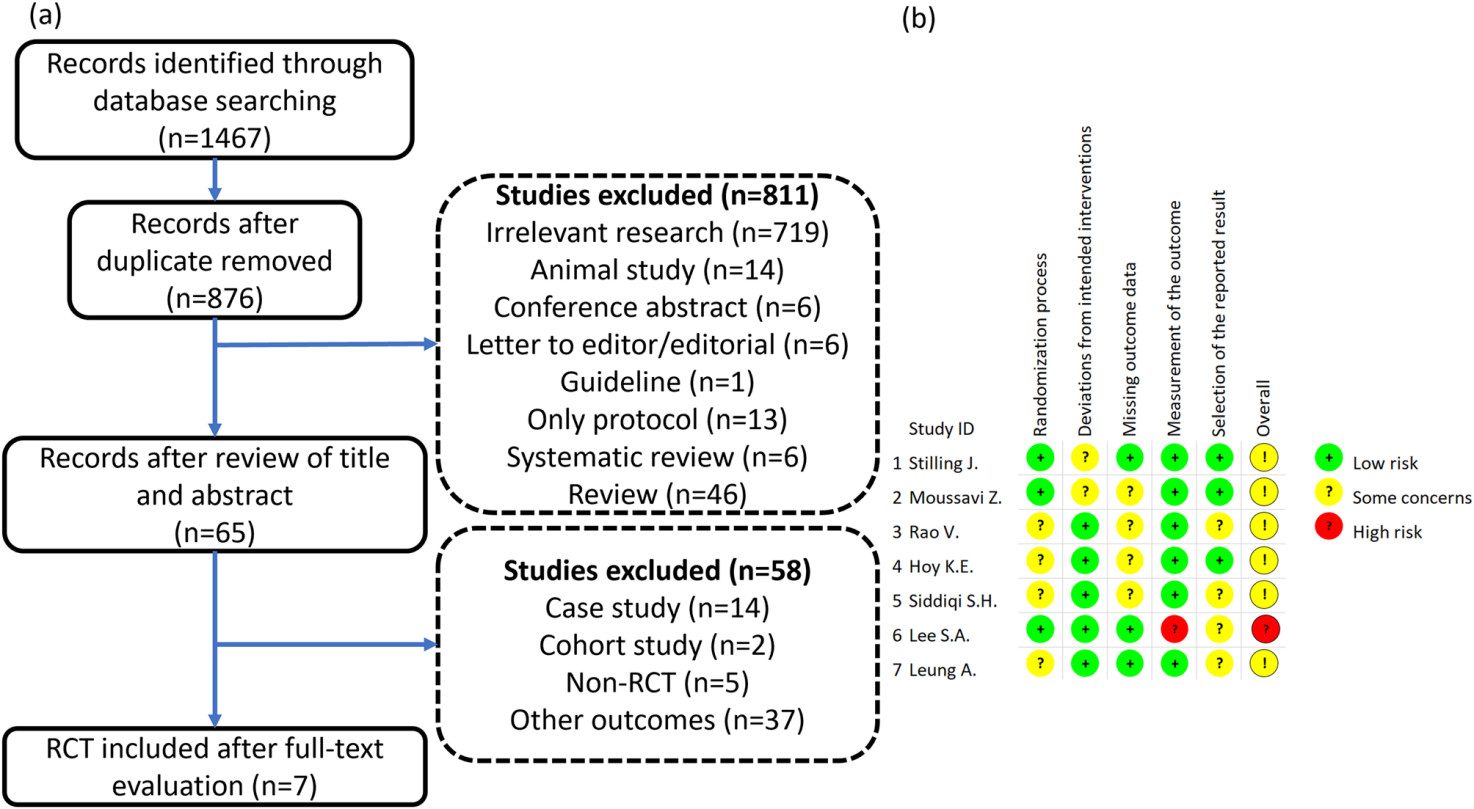
Flowchart of study selection and risk of bias of included studies. (**a**) Flowchart and (**b**) risk of bias.

### Study characteristics

Table 1 presents the characteristics of the selected studies. In total, 136 participants were included in our research; 79 participants who had experienced TBI and had post-TBI depression were included in four studies; 57 participants with a diagnosis of posttraumatic headache or PCS had moderate to severe depression on average in three studies. The participants in the included articles had a mean age of 34.1–46.3 years and a higher percentage of men (73; 54%) than women. The rTMS protocols were summarized in Table 2.

**Table 1:** Summary of study characteristics and outcomes measured.

Author (year)	Patient population	Mean age	Men	Women	Number of participants in the control and intervention groups	Study design	Outcomes measured and parameters used		
							Depression average score (baseline)	Cognition	Postconcussion syndrome
Stilling (2020)	Mild TBI with PTH and PPCS	36	2	18	Sham rTMS N = 10 Real rTMS N = 10	Double-blinded RCT	PHQ-9 11.9	–	RPQ-3, RPQ-13
Moussavi (2019)	*TBI with PPCS	45.9	2	6	Sham rTMS N = 4 Real rTMS N = 4	Double-blinded RCT	MADRS 19.65	–	RPQ-3, RPQ-13
Rao (2019)	TBI with depression	40.0	16	14	Sham rTMS N = 17 Real rTMS N = 13	Double-blinded RCT	HAMD 23.5	TMT-A, TMT-B, BVMT, SCWT	RPQ-13
Hoy (2019)	TBI with moderate to severe depression	46.3	10	11	Sham rTMS N = 10 Real rTMS N = 11	Double-blinded RCT	MADRS 34.0	TMT-A, TMT-B, BVMT, SCWT	-
Siddiqi (2019)	Moderate TBI with treatment-resistant depression	45.8	11	4	Sham rTMS N = 6 Real rTMS N = 9	Double-blinded RCT	MADRS 32.2	–	–
Lee (2018)	TBI with mild to moderate depression	41.9	9	4	NDT + Sham rTMS N = 6 NDT + Real rTMS N = 7	Single-blinded RCT	MADRS 23.8	TMT, SCWT	–
Leung (2018)	Mild TBI with PTH	34.1	23	6	Sham rTMS N = 15 Real rTMS N = 14	Double-blinded RCT	HAMD 23.9	–	–

*TBI* traumatic brain injury, *PTH* posttraumatic headache, *PPCS* persistent postconcussion syndrome, *NDT* neurodevelopmental therapy, *rTMS* repetitive transcranial magnetic stimulation, *RPQ* Rivermead Post-Concussion Symptom Questionnaire, *MADRS* Montgomery–Asberg Depression Rating Scale, *HAMD* Hamilton Rating Scale for Depression, *PHQ-9* Patient Health Questionnaire 9, *TMT* Trail Making Test, *BVMT* Brief Visuospatial Memory Test, *SCWT* Stroop Color Word Test, *RCT* randomized controlled trial.

*Only people having severe postconcussion syndrome (SPCS) were included according to inclusion criteria.

**Table 2:** Summary of rTMS protocols in included studies.

Author/year	Coil type	Location	Frequency (Hz)	Intensity (% RMT)	Sessions	Pulses × train(s)/session	Duration of each train (s)	Intertrain interval (s)
Stilling (2020)	Figure of 8 coil	Left DLPFC	10	70	10	60 × 10	6	45
Moussavi (2019)	Figure of 8 coil	Left DLPFC	20	100	13	30 × 25	1.5	10
Rao (2019)	Figure of 8 coil	Right DLPFC	1	110	20	300 × 4	300	60
Hoy (2019)	Figure of 8 coil	Bilateral sequential DLPFC (right → left)	Right: 1 Left: 10	110	20	Right: 900 × 1 Left: 50 × 30	5	Right: nil Left: 25
Siddiqi (2019)	NA	Bilateral sequential DLPFC (left → right)	Right: 1 Left: 10	120	20	Right: 1000 × 1 Left: 50 × 80	5	Right: nil Left: 20
Lee (2018)	Figure of 8 coil	Right DLPFC	1	100	10	40 × 50	40	25
Leung (2018)	Figure of 8 coil	Left DLPFC	10	80	4	100 × 10	10	1

*DLPFC* dorsolateral prefrontal cortex, *RMT* resting motor threshold.

### Measured outcomes

#### Depression

Four studies reported depression severity according to the MADRS^16,26–28^, and two articles used the HAMD to assess depression^29,30^. Another study by Stilling et al. measured the degree of depression by using the Patient Health Questionnaire 9 (PHQ-9)^31^. By using the mean values of baseline depression severity score, six studies [Hoy et al. (MADRS = 34.0), Siddiqi et al. (MADRS = 32.2), Lee et al. (MADRS = 23.8), Rao et al. (HAMD = 23.5), Leung et al. (HAMD = 23.9), and Stilling (PHQ-9 = 11.9)] enrolled participants with moderate to severe depression (MADRS > 19, HAMD > 13, and PHQ-9 > 9)^16,27–31^. A study by Moussavi et al. recruited participants with light PCS (LPCS) and severe PCS (SPCS). Participants with LPCS and SPCS had mild depression (MADRS = 10.2) and moderate to severe depression (MADRS = 19.65), respectively^26^. Given the inclusion criteria of the present study, only participants with moderate to severe depression were included.

#### Cognition and attention

Three studies used the Trail Making Test (TMT), a neuropsychological test of processing speed, attention, and task shifting to examine cognitive function. The TMT can be further divided into TMT-A and TMT-B. In TMT-A, participants must connect 25 circles numbered 1–25 in numerical order. In TMT-B, participants must alternate between numbers (1–13) and letters (A to L) in an ascending order (1-A-2-B-3-…)^32^. Two studies examined cognitive function by using TMT-A and TMT-B^27,29^, whereas another study by Lee et al. evaluated the outcome by summing the time required for TMT-A and TMT-B^28^.

The Stroop Color Word Test (SCWT) is a test of selective attention. A conflict is created through presenting a mismatch between the name of a color (“green”) and the color of the printed word (e.g., the word green printed using red ink)^33^. To examine the ability to overcome the cognitive interference, the time required for the SCWT was measured in three studies^27–29^. In the Brief Visuospatial Memory Test (BVMT), the participants were asked to memorize and reproduce six types of shapes^34^. After three learning trials, the scores of all rounds were summed (max = 2 × 6 × 3 = 36; 2 points for each figure in three trials). Two articles used the BVMT ^27,29^.

#### Postconcussion syndrome

Three studies recruited patients with PCS evaluated by using the self-reported Rivermead Post-Concussion Symptom Questionnaire (RPQ-3 and RPQ-13)^26,29,31^. The RPQ-3 inquires into the symptoms of headache, nausea/vomiting, and dizziness, three early postconcussion symptoms. The RPQ-13 questionnaire inquires into 13 self-reported conditions, namely noise sensitivity, sleep disturbance, fatigue, being irritable, feeling depressed, feeling frustrated, forgetfulness, poor concentration, taking longer to think, blurred vision, light sensitivity, double vision, and restlessness, to measure cognitive and emotional symptoms^35^.

### Study quality

The baseline imbalance of patient characteristics in three articles^16,27,30^ and unclear allocation concealment in Rao et al. resulted in a risk of uncertainty bias in the randomization process. Because depression improvement could confound the measurement of somatic symptoms (i.e., PCS), such deviations may have increased bias risk in outcome assessment in two studies^26,31^. Four articles had risk of bias due to high drop-out or loss-to-follow-up rates (> 5%)^16,26,27,29^. The study by Lee et al. had a high risk of bias of outcome measurement owing to unblinded assessors^28^. Trials that were not analyzed according to prespecified protocols in four studies (Rao et al., Siddiqi et al., Lee et al., and Leung et al.) led us to identify a potential risk of reporting bias^16,28–30^ (Fig. 1b).

### Effectiveness

#### Depression

Five studies evaluated the effect of rTMS treatment on depression and measured depression outcome immediately after rTMS treatment^16,26–28,30^. In a random-effects model immediately after rTMS intervention, rTMS had a high mean effect size of 1.03 (95% confidence interval [CI] 0.20–1.86) without heterogeneity (I^2^ = 0%; Fig. 2a). However, 1 month after rTMS treatment, the effect size of rTMS became smaller and statistically nonsignificant (effect size = 0.39; 95% CI − 1.15 to 1.93; I^2^ = 0%), although only three studies were included in the analysis^26,29,31^ (Fig. 2b).

**﻿Figure 2:**
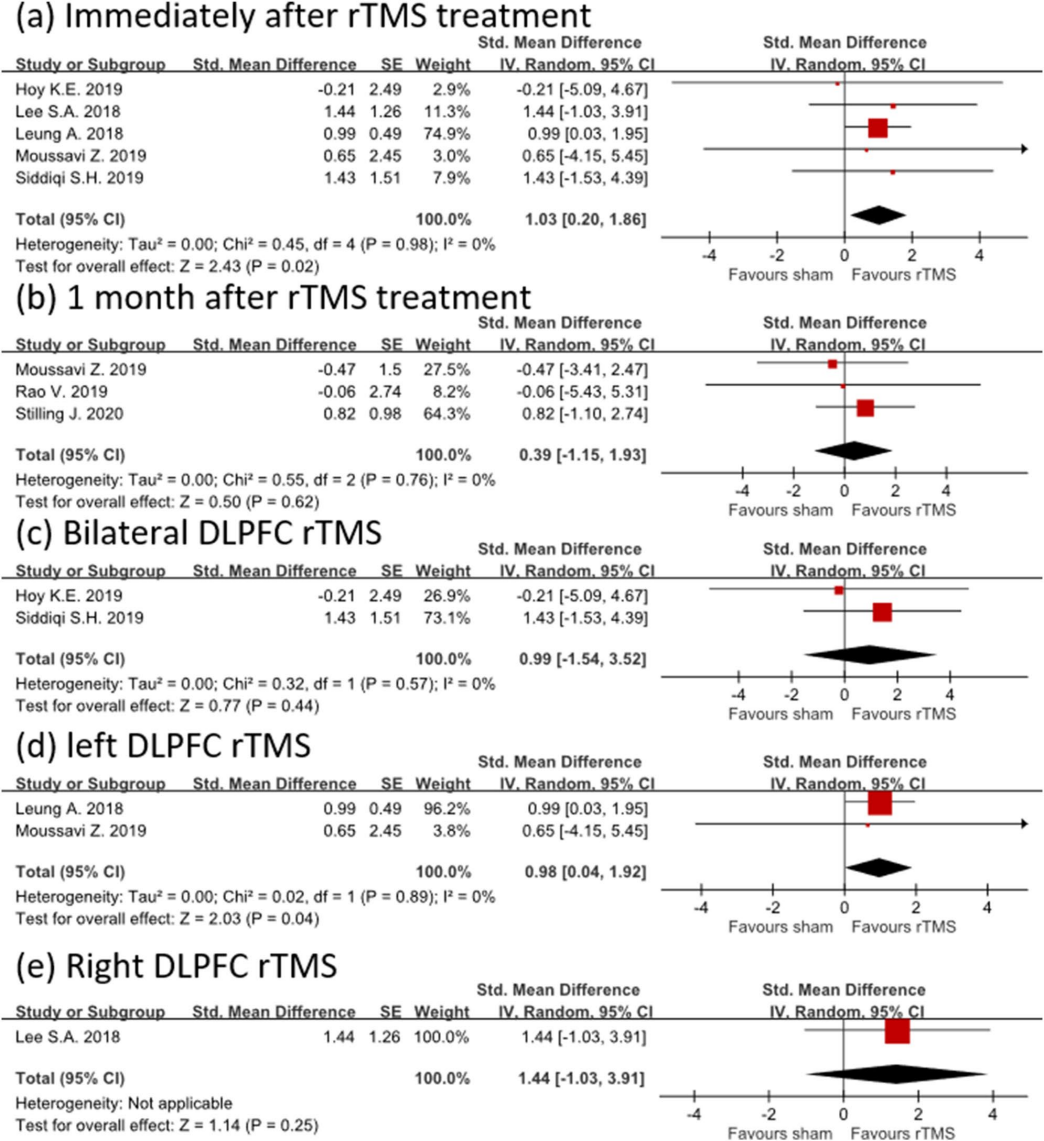
Forest plot showing the efficacy of rTMS versus sham in treating depression. The depression outcome was assessed (**a**) immediately and (**b**) 1 month after rTMS treatment. In the subgroup analysis, the depression outcome was measured immediately after (**c**) bilateral, (**d**) left, and (**e**) right rTMS treatment.

In the subgroup analysis, we further analyzed the effect of rTMS on depression immediately after intervention according to different stimulation location. We observed that the results of bilateral sequential DLPFC rTMS therapy had an effect size of 0.99 in treating post-TBI depression, but these results failed to reach statistical significance when compared with the sham group (95% CI  − 1.54 to 3.52; I^2^ = 0%; Fig. 2c). By contrast, left DLPFC rTMS treatment of post-TBI depression exhibited significant effectiveness compared with the sham group (effect size = 0.98; 95% CI 0.04–1.92; I^2^ = 0%) despite having an effect size similar to bilateral sequential DLPFC rTMS (Fig. 2d). Only one study investigated the efficacy of right DLPFC rTMS intervention in post-TBI depression. Their data revealed that right DLPFC rTMS had a large effect size (1.44) after right DLPFC rTMS treatment although it did not reach statistical significance (CI − 1.03 to 3.91; Fig. 2e).

#### Cognition and attention

The mean effect sizes of rTMS on processing speed were − 0.25 (CI − 0.62 to 0.12; I^2^ = 0%) for TMT-A and − 0.26 (CI − 1.70 to 1.18; I^2^ = 0%) for TMT-B. When both TMT-A and TMT-B were synthesized, the effect size was − 0.18 (CI − 0.53 to 0.17; I^2^ = 0%). All the results favored the sham group but were not statistically significant (Fig. 3a–c).

**﻿Figure 3:**
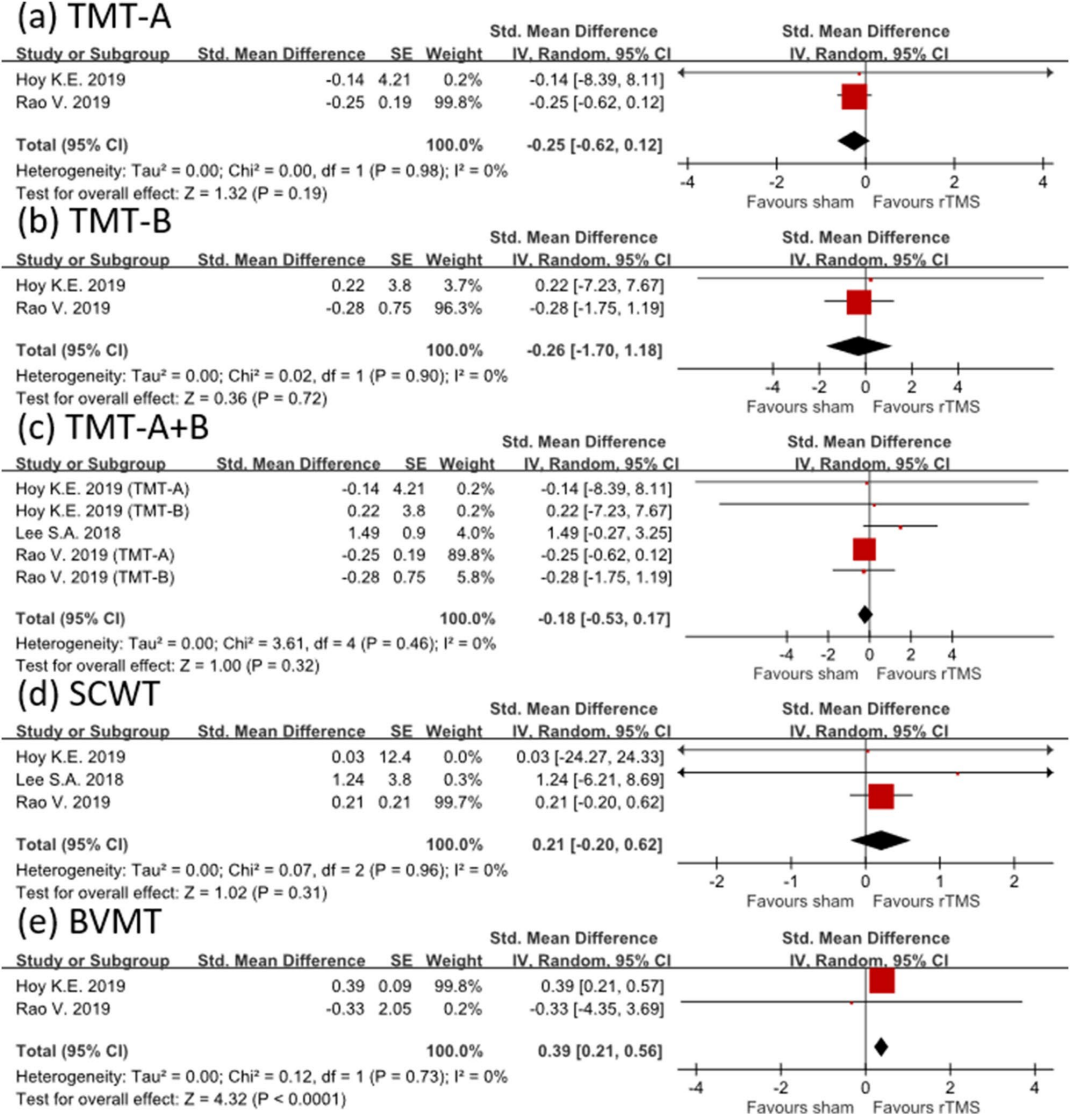
Forest plot showing efficacy of treatment versus sham treatment in improving cognitive function. Different cognitive domains, including (**a**–**c**) processing speed, (**d**) selective attention, and (**e**) visuospatial memory, were evaluated.

Three studies assessed the effectiveness of rTMS treatment in improving selective attention by using the SCWT. The effect size after rTMS intervention was small (effect size = 0.21; 95% CI − 0.20 to 0.62; I^2^ = 0%; Fig. 3d). These results indicated that the efficacy of rTMS therapy in restoring selective attention was limited. However, rTMS significantly improved visuospatial memory, with an effect size of 0.39 (CI 0.21–0.56; I^2^ = 0%; Fig. 3e).

#### Postconcussion syndrome

The effect of rTMS on PCS was small (effect size = 0.34; 95% CI  − 0.34 to 1.02; I^2^ = 0%) based on RPQ-3 assessment. Moreover, the rTMS group did not exhibit a favorable outcome relative to the control group (effect size =  − 0.5; 95% CI − 0.97 to − 0.04; I^2^ = 0%) when RPQ-13 was used to examine the effectiveness of rTMS intervention (Fig. 4a, b).

**﻿Figure 4:**
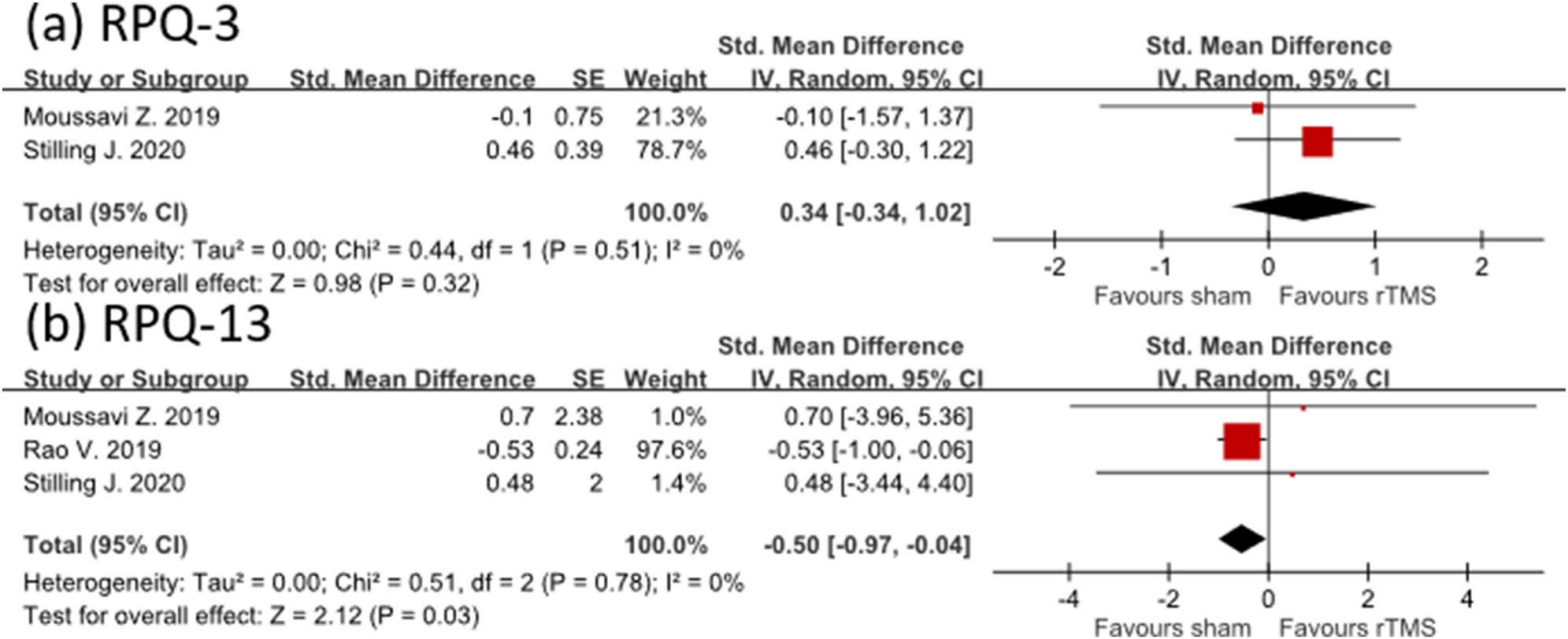
Forest plot showing efficacy of treatment versus sham treatment in postconcussion syndrome. (**a**) RPQ-3 and (**b**) RPQ-13.

## Discussion

Several studies have attempted to use rTMS to enhance the recovery of individuals with post-TBI depression. However, insufficient evidence was discerned regarding the effect of TMS on people with post-TBI depression. Hence, this study was designed to investigate the effectiveness of rTMS in treating post-TBI depression. Our results demonstrated that rTMS has a significant positive effect on post-TBI depression immediately after administration. Nevertheless, the effect size decreased over time. We observed no significant effect 1 month after rTMS treatment compared with the sham group. However, sample sizes of previous studies were small. Moreover, few studies have performed a follow-up assessment, making it difficult to determine long-term efficacy (e.g., 4 weeks) of rTMS intervention in the present study. In addition, MDD has 80% relapse rate after acute treatment of rTMS^36^ and the reintroduction of rTMS is needed in 36.2% depressed patients without TBI^37^. Nevertheless, the trial for maintenance rTMS therapy in individuals with post-TBI depression is still lacking. Therefore, developing protocols of maintenance TMS therapy^38^ as well as recruiting a sufficient number of participants and having a long follow-up period in future studies are essential.

Relevant studies have proved that both unilateral and bilateral rTMS are effective in individuals with depression. Moreover, their results did not conclude that bilateral rTMS was superior to unilateral stimulation in remitting TRD^39^. A study by Trevizol et al. revealed that bilateral rTMS was superior to unilateral in elderly individuals with TRD^40^. Subgroup analysis in the present study indicated that both left and bilateral rTMS had a large effect size, although the effect of bilateral rTMS did not reach statistical significance. Notably, however, Siddiqi et al.^16^ determined that bilateral rTMS had a large effect size on TRD in patients with TBI. Thus, their data indicated that bilateral rTMS could play a promising role in treating depression in people with TBI.

A systematic review by Martin et al. revealed that applying rTMS to the prefrontal cortex of patients with depression modestly enhanced the cognition of these patients. Furthermore, such an effect was independent of the antidepressant effect of rTMS^41^. However, the studies included in the present study did not indicate that rTMS therapy significantly contributed to improvement in cognition and PCS in patients with TBI. A prospective, non-randomized trial, which enrolled patients with PCS (60% having depression), demonstrated significant improvement in SCWT and PCS scores but not for TMT scores^42^. Our analysis indicated that brief visuospatial memory (BVMT) but not processing speed (TMT) or PCS improved after rTMS treatment. The rTMS treatment for the inhibitory ability of cognitive interference (SCWT) was borderline effective. In a systemic review evaluating the cognitive effect of HF rTMS on depression in patients, few studies in their review demonstrated significant cognition-enhancing effects of rTMS, including selective attention (3 of 9), spatial memory (1 of 6), and processing speed (4 of 13)^43^. Moreover, another meta-analysis demonstrated a modest effect of rTMS on cognition in patients with depression and a significant improvement only in TMT-A and TMT-B^41^. The present results differ from those of previous investigations on the effect of rTMS on cognition and PCS. One of the reasons for this may be that previous studies and our study included different populations in the analysis. Furthermore, the parameters of rTMS treatment and the testing of outcome variables were different among previous studies and ours. However, further evaluation of the effectiveness of rTMS on cognition and PCS in patients with TBI is necessary. In addition, cognitive function consists of several domains that involve different cortex areas in the cognitive control network^13^; the pathophysiology of cognitive enhancement by applying rTMS to patients with TBI should be further investigated.

Establishing and developing a universal protocol of rTMS administration (for example, with respect to duration, number of sessions, and dosage of rTMS application) is essential for improving the depression status and cognitive function of individuals with TBI. A minimum of 4-week (20 sessions) rTMS treatment was needed for cognitive enhancement in patients with psychiatric problems^44^. Moreover, a randomized controlled study revealed that 8 weeks are required for rTMS to improve cognitive function, including memory, attention, visuospatial function, and language in patients with schizophrenia^45^. Teng et al. suggested that left HF rTMS with 1200–1500 pulses/day with 20 sessions exerted the best antidepressant effect^46^, and another study found that cumulative sessions rather than pulses determined the recovery rate in major depression^47^. The present meta-analysis demonstrated that previous studies used heterogeneous protocols, including number of sessions (4–20 times), total number of pulses (600–4000 times/day), application frequency, and stimulation intensity. Yet, anatomic variation between subjects may also have contributed to the heterogeneity of the results. TBI complications vary from patient to patient due to the highly heterogenous nature of the disease with various injury mechanisms^48^. This variability may result from the disruption of different brain networks underlying the mechanism of post-TBI complications. Therefore, stimulation protocols—for example, location, frequency and intensity of stimulation, total number of pulses, as well as total amount of sessions—must be further determined.

This meta-analysis has several limitations. First, most of the included studies had a small sample size. Small sample size leads to low statistical power and subsequently limits the reliability of the results regarding the therapeutic effectiveness of rTMS in treating depression and cognitive impairment in patients following TBI. Second, although all the studies had applied rTMS to the left DLPFC area of the brain, stimulation protocols, including rTMS intervention duration, number of sessions, number of pulses, and stimulation intensity, varied. Thus, efficacy assessment and reproducibility of their investigations are difficult. Third, as few studies have executed a 1-month follow-up, determining the long-term effect of rTMS on depression and cognition of patients with TBI is difficult. Fourth, due to a lack of a mixed model design, determining the effect of rTMS on cognitive impairment is difficult in patients with TBI with concomitant depression status. Finally, several studies found variation in network topography between individuals, and image- or navigation-guided rTMS therapy may be considered in future work.

## Conclusion

Our study demonstrated that rTMS has a short-term effect on post-TBI depression, whereas the effects of rTMS on cognitive impairment and PCS in patients with TBI remained inconclusive. In addition, RCT studies revealed that rTMS has a significant antidepressant effect only when applied to the left DLPFC area. However, previous studies were limited by small sample sizes and heterogenous methodologies. Further large sample sizes and well-designed trials are necessary to confirm the antidepressant and cognition-enhancing effects of rTMS and to establish optimal stimulation protocols for administering rTMS to patients with post-TBI depression.
